# Development of a Chemoresistant Risk Scoring Model for Prechemotherapy Osteosarcoma Using Single-Cell Sequencing

**DOI:** 10.3389/fonc.2022.893282

**Published:** 2022-05-18

**Authors:** Ziliang Zeng, Wenpeng Li, Di Zhang, Chi Zhang, Xu Jiang, Rui Guo, Zheyu Wang, Canchun Yang, Haolin Yan, Zhilei Zhang, Qiwei Wang, Renyuan Huang, Qiancheng Zhao, Bo Li, Xumin Hu, Liangbin Gao

**Affiliations:** Department of Orthopedics, Sun Yat-sen Memorial Hospital, Guangzhou, China

**Keywords:** osteosarcoma, single-cell RNA sequencing, chemoresistance, heterogeneity, stemness

## Abstract

**Background:**

Chemoresistance is one of the leading causes that severely limits the success of osteosarcoma treatment. Evaluating chemoresistance before chemotherapy poses a new challenge for researchers. We established an effective chemoresistance risk scoring model for prechemotherapy osteosarcoma using single-cell sequencing.

**Methods:**

We comprehensively analyzed osteosarcoma data from the bulk mRNA sequencing dataset TARGET-OS and the single-cell RNA sequencing (scRNA-seq) dataset GSE162454. Chemoresistant tumor clusters were identified using enrichment analysis and AUCell scoring. Its differentiated trajectory was achieved with inferCNV and pseudotime analysis. Ligand–receptor interactions were annotated with iTALK. Furthermore, we established a chemoresistance risk scoring model using LASSO regression based on scRNA-seq-based markers of chemoresistant tumor clusters. The TARGET-OS dataset was used as the training group, and the bulk mRNA array dataset GSE33382 was used as the validation group. Finally, the performance was verified for its discriminatory ability and calibration.

**Results:**

Using bulk RNA data, we found that osteogenic expression was upregulated in chemoresistant osteosarcoma as compared to chemosensitive osteosarcoma. Then, we transferred the bulk RNA findings to scRNA-seq and noticed osteosarcoma tumor clusters C14 and C25 showing osteogenic cancer stem cell expression patterns, which fit chemoresistant characteristics. C14 and C25 possessed bridge roles in interactions with other clusters. On the one hand, they received various growth factor stimulators and could potentially transform into a proliferative state. On the other hand, they promote local tumor angiogenesis, bone remodeling and immunosuppression. Next, we identified a ten-gene signature from the C14 and C25 markers and constructed a chemoresistant risk scoring model using LASSO regression model. Finally, we found that chemoresistant osteosarcoma had higher chemoresistance risk score and that the model showed good discriminatory ability and calibration in both the training and validation groups (*AUC_train_* = 0.82; *AUC_valid_* = 0.84). Compared with that of the classic bulk RNA-based model, it showed more robust performance in validation environment (*AUC_valid-scRNA_ =* 0.84; *AUC_valid-bulk DEGs_* = 0.54).

**Conclusions:**

Our work provides insights into understanding chemoresistant osteosarcoma tumor cells and using single-cell sequencing to establish a chemoresistance risk scoring model. The model showed good discriminatory ability and calibration and provided us with a feasible way to evaluate chemoresistance in prechemotherapy osteosarcoma.

## Introduction

Osteosarcoma is the most common malignant bone tumor, primarily threatening children and adolescents. The present treatment strategy for primary osteosarcoma mainly consists of local resection and systematic chemotherapy (1). Unfortunately, the prognosis of osteosarcoma patients remains unsatisfactory, and the 5-year overall survival (OS) rate of osteosarcoma patients who receive complete resection and standard chemotherapy is approximately 70% (2). The major problem that severely limits the success of osteosarcoma treatment is a poor histopathologic response to neoadjuvant chemotherapy, which increases the risk of developing metastasis and relapse (3, 4). However, the histopathologic response cannot be evaluated until tissue is obtained during surgery; thus, timely adjustment of neoadjuvant chemotherapy to overcome drug resistance is difficult. Evaluating chemoresistance before chemotherapy poses a new challenge for researchers.

Researchers have continuously explored prechemotherapeutic methods for predicting the osteosarcoma response to chemotherapy. The application of evolutionary theory to cancer provides the groundwork for forecasting histopathologic response before chemotherapy in osteosarcoma (5). Theoretically, the chemotherapeutic environment puts selection pressure on tumor cell pools and causes existing chemoresistant cells to become the dominant population. In osteosarcoma, previous studies also strongly support the idea that chemoresistance results from the population of chemoresistant cells (6). Early detection of chemoresistant cells in biopsy can indicate an increased risk of drug resistance (7). Bulk RNA sequencing (RNA-seq) is being increasingly recognized as a feasible method to identify chemoresistant cells in biopsy tissue (8). RNA-seq can help researchers identify various chemoresistance-related biomarkers and thus select existing relevant therapeutic agents, which is a feasible way to improve the survival rate for osteosarcoma (6). However, RNA-seq resolves the average gene expression of bulk tissue and weakens its detection of the expression patterns of small groups among environmental noise.

**Figure 1:**
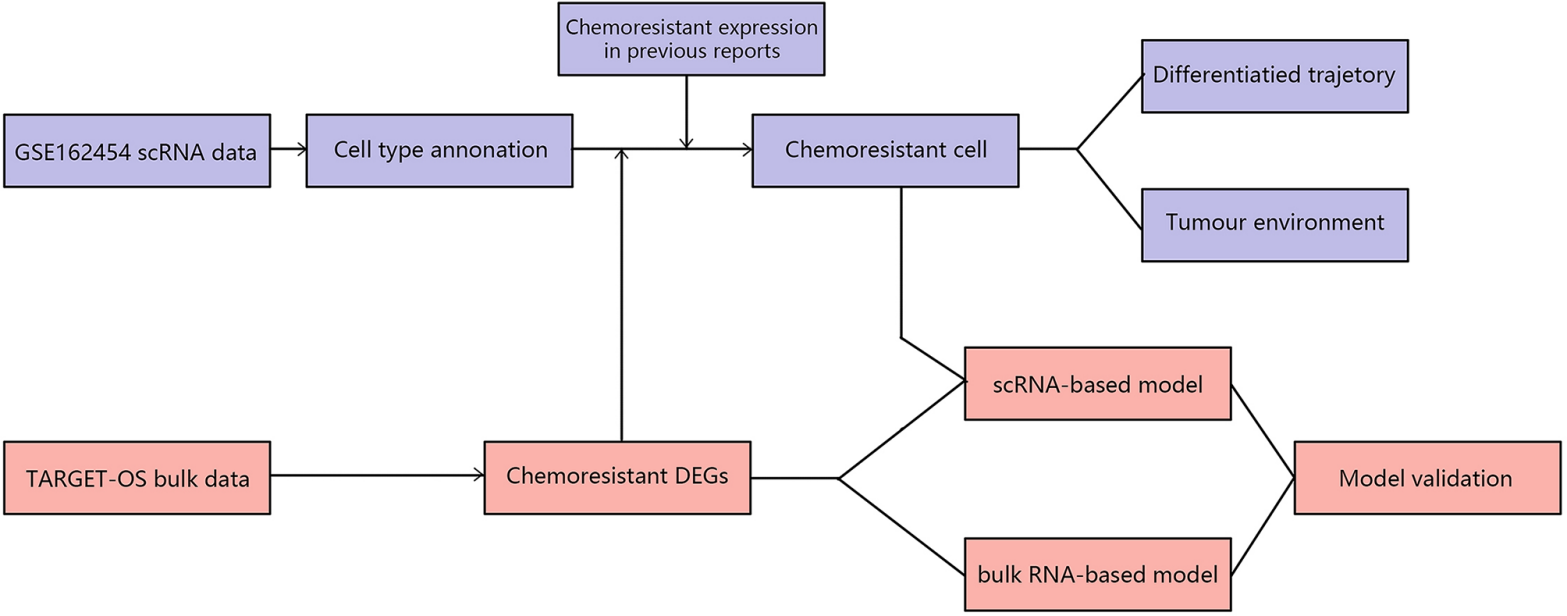
Design of the experiment and workflow of this study.

Made available with technological advances, single-cell RNA sequencing (scRNA-seq) can resolve gene expression at the individual cell level and enable us to better explore and identify chemoresistant cells. Liu Y’s and Zhou Y’s first applied scRNA-seq to reveal the landscapes of tumor environments in osteosarcoma (9, 10). Comparing the two techniques, scRNA-seq has higher sensitivity but higher cost, whereas bulk RNA-seq has more samples and reliably reflects the characteristics among populations. Thus, utilizing the connection between scRNA and bulk RNA-seq data could be instrumental in understanding and recognizing tumor chemoresistance in osteosarcoma. This study focused on comprehensively analyzing prechemotherapy osteosarcoma by combining bulk RNA-seq and scRNA-seq to reveal chemoresistant osteosarcoma tumor cell expression patterns and their role in interactions with other cellular components in the osteosarcoma environment and to establish a *chemoresistance risk scoring model* in prechemotherapy osteosarcoma. First, we revealed the general glance in chemoresistant osteosarcoma based on bulk RNA-seq data. Second, we identified chemoresistant tumor cells based on combining bulk RNA results and existing chemoresistant markers (6). Third, we undertook a more thorough analysis of chemoresistant cells in tumor cells and tumor environments. Finally, chemoresistance-related expression patterns were utilized to establish a *chemoresistance risk scoring model* for prechemotherapy osteosarcoma. The workflow in our study is summarized in **Figure 1**.

## Material and Methods

### Data Sources

The data sources in our study are summarized in **Table 1**. The bulk RNA-seq data were obtained from the Therapeutically Applicable Research to Generate Effective Treatments program: Osteosarcoma (TARGET-OS) (phs000468; https://ocg.cancer.gov/programs/target/projects/osteosarcoma) (11). The enrolled patients had 1. available mRNA sequencing data from osteosarcoma samples taken before chemotherapy and 2. complete clinical data and follow-up records, including age, sex, metastatic stage at diagnosis, and tumor response to chemotherapy (necrotic rate ≥90% or Huvos stage III & IV indicated chemosensitive osteosarcoma; necrotic rate <90% or Huvos stage I & II indicated chemoresistant osteosarcoma). The data were transformed into transcripts per kilobase million (TPM) values, and *log*_2_(*TPM*+1) values were calculated before further analysis.

**Table 1:** Data sources.

Project	Datasets	Sample size
DEGs^†^ analysis using bulk RNA-seq^‡^	TARGET-OS (11)	43
**Inclusion criteria:**
1. Available mRNA sequencing data from osteosarcoma samples taken before chemotherapy; 2. Complete clinical data and follow-up records, including age, sex, metastatic stage at diagnosis, and tumor response to chemotherapy.
Single cell analysis using scRNA-seq^§^	GSE162454 (9)	6
**Inclusion criteria:**
Available scRNA sequencing data from osteosarcoma samples taken before chemotherapy;
Establishment and validation of the chemoresistance risk model		
Training group	TARGET-OS (11)	29
Validating group	GSE33382 (12)	31
**Inclusion criteria:**
The same as DEGs analysis section
**Exclusion criteria:**
1. No distal metastasis at diagnosis (Enneking IIb); 2. Available records, including tumor progression and progression time for PFS^¶^.

^†^DEG is “Differentially expressed genes”.

^‡^RNA-seq is “RNA sequencing”.

^§^scRNA-seq is “Single-cell sequencing”.

^¶^PFS is “Progression-free survival”.

scRNA sequencing data were obtained from *GSE162454* (https://ftp.ncbi.nlm.nih.gov/geo/series/GSE162nnn/GSE162454/) from the *Gene Expression Omnibus (GEO) database* (9). *GSE162454* data were collected from conventional osteosarcoma biopsy samples taken before chemotherapy, and OS1-6 cases were included in our study. The osteosarcoma single-cell suspension was loaded onto a *10x Genomics Chromium Single-Cell Chip* and sequenced on an *Illumina HiSeq X Ten instrument*.

In the “Establishment and validation of the chemoresistance risk model” section, the *TARGET-OS* series was used as the training set, and *GSE33382* (https://ftp.ncbi.nlm.nih.gov/geo/series/GSE33nnn/GSE33382/matrix/) was used as the validating set (11, 12). The enrolled patients were not only eligible according to the above standards but also had 1. no metastasis at diagnosis and 2. available follow-up records, including tumor progression and progression time for progression-free survival (PFS). *GSE33382* is RNA array data on the *Illumina human-6 v2.0 expression beadchip* platform. The batch effect between *TARGET-OS* and *GSE33382* was adjusted based on *empirical Bayes frameworks* using the *sva* package (13).

### Bulk RNA-Seq Expression Profiling of Chemoresistant Osteosarcoma Patients

We used the *EdgeR* package to identify differentially expressed genes (DEGs) (14). We kept those genes with average expression>1. The *p* values were adjusted using the *Benjamin* & *Hochberg* method, and the cutoff value was less than 0.05. The absolute cutoff *logFC* value was more than 0.25. The upregulated and downregulated DEGs were subjected to enrichment analysis using *Metascape (* 15). In the enrichment analysis, an adjusted *p* value <0.01, *q-*value <0.05 and minimum enrichment score >1.5 were set as the cutoff criteria.

### scRNA-Seq Data Processing and Cell Annotation

The scRNA-seq data were processed with the *Seurat* package (16). Individual data were merged, and low-quality cells were excluded based on the types of genes detected, total number of detected genes, and percentage of mitochondrial genes. The eligible data were normalized, and batch effects were removed. Furthermore, the scRNA-seq data were subjected to *uniform manifold approximation and projection* (UMAP) analysis for dimension reduction and statistically divided into different clusters with a resolution of 1.0. Using the *FindAllMarkers* function, we obtained markers among different clusters and annotated their cell types based on known cell markers.

### Determination of the Chemoresistant Expression Profile in Tumor Cells

We filtered the chemoresistant clusters according to the chemoresistance-related expression profile in the tumor cells. Chemoresistant expression was evaluated from 2 perspectives. First, based on bulk RNA results, we focused on osteoblastic lineage expression, including cell proliferation, extracellular matrix (ECM) secretion, and ossification induction among tumor cells (17). Markers (top 100 genes) from each tumor cell cluster were subjected to enrichment analysis using *Metascape (* 15). The parameter settings are the same as those mentioned above.

Second, we collected chemoresistance-related expression based on previous reports and included the ABC transporter gene set, DNA repairment gene set, and stemness-related gene sets (EMT gene set, Wnt/β-catenin gene set, TGFβ gene set, TNFα gene set, MAPK gene set, Notch gene set, Hedgehog gene set and BMP gene set) (6). Due to the inherent sparsity of single-cell data, the scRNA-seq data were transformed into *pseudobulk* profiles for each tumor before comparison with *AUCell*, which identifies the active state of gene sets in scRNA-seq data (18). The high *AUCell* score cells were divided by AUC cutoff values.

### Differentiation Trajectory Analysis of Chemoresistant Tumor Cells

The chemoresistant tumor cells and the remaining tumor cells from the same patients were subjected to differentiation trajectory analysis. It was achieved with 2 steps. First, we estimated the DNA variation to judge the degree of differentiation; second, we performed *pseudotime* analysis to explore the cell-state transitions.

Tumor cell differentiation is an important resource of tumor diversity, and DNA variation could infer the degree of differentiation, in which higher DNA variation indicates a higher differentiation degree (19). We estimated the chromosomal copy number variation (CNV) by the *inferCNV* package (20). The *hidden Markov model* (HMM) was utilized to minimize noise, and the annotated immune cells were used as a normal cell control. The cutoff value for the minimum average read counts per gene among reference cells was set as 0.1. The CNV score was calculated as the mean of the CNV regions. Then, based on the CNV results, *pseudotime* analysis was utilized to test cell state transmission with the *Monocle2* package (21). It was used to calculate differentiation-related gene expression and visualize the differentiatied trajectory tree of chemoresistant tumor cells.

### Tumor Environment Evaluation of Chemoresistant Tumor Cells

We focused on 2 aspects of the tumor environment surrounding chemoresistant tumor cells. First, we undertook a more thorough analysis in nontumor cell clusters. We rearranged their markers and annotated them with reported tumor-related subtype markers. Second, *ligand–receptor (LR) interaction* analysis was applied to demonstrate the interaction between the tumor and environment using the *iTALK* package (22). The ligand–receptor interaction of interest was assessed based on the expression of the ligand in the CRC cluster and the expression of the corresponding receptor in another cell cluster. Only the top 20 expressed genes and markers expressed in the corresponding cell types were considered in the analysis. Then, we selected the LR interactions that were biologically relevant to the osteosarcoma environment.

### Construction and Validation of a Chemoresistance Risk Scoring Model of Prechemotherapy Osteosarcoma

As mentioned above, the TARGET-OS series was used as the training set, and GSE33382 was used as the validating set (11, 12). The gene markers of chemoresistant tumor cells based on scRNA-seq were a pool of candidate predictors. First, univariate analysis was applied to narrow down the candidate predictors as upregulated genes in chemoresistant osteosarcoma. Second, *least absolute shrinkage and selection operator* (*LASSO*) analysis was utilized to detect predictors using the *glmnet* package (23). LASSO analysis was repeated over 100 iterations to fit the optimal model. Third, these predictors were utilized to develop the *binary logistic regression* model for scoring chemoresistance risk in osteosarcoma. The chemoresistant risk score was calculated with the following formula: $Y=\sum_{i=1}^{n}coef_{i}*X_{i}$ , where *“coef_i_”* and *“X”* denote the coefficient and expression level of each predictor. Ultimately, a *receiver operating characteristic (ROC)* curve was utilized to examine the performance of the predictive model, and an *area under the curve (AUC)* value of >0.80 indicated good performance.

## Result

### Identification of DEGs in Chemoresistant Osteosarcoma Using Bulk RNA-Seq

**Figure 2:**
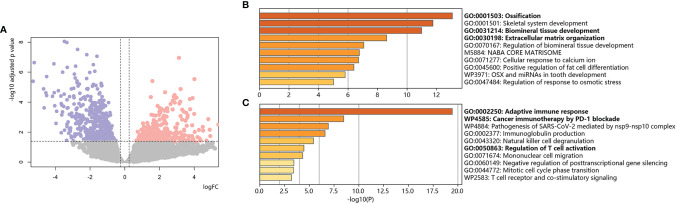
Bulk RNA-based chemoresistant osteosarcoma expression patterns. **(A)** Volcano plot of DEGs in chemoresistant osteosarcoma versus control. Red dots indicate upregulated genes, blue dots indicate downregulated genes, and gray dots indicate genes without significant changes. The dotted line shows the cutoff values for adjusted p values <0.05 & absolute value of logFC >0.25. **(B)** Bar plot for the top-ranked gene set enrichment in upregulated DEGs in chemoresistant osteosarcoma. **(C)** Bar plot for the top-ranked gene set enrichment in downregulated DEGs in chemoresistant osteosarcoma.

We employed 43 primary osteosarcoma bulk RNA-seq samples from TARGET-OS, 25 samples from chemoresistant patients and 18 samples from chemosensitive patients (**Table 2**) (11). We identified 111 upregulated DEGs and 245 downregulated DEGs in chemoresistant patients compared with chemosensitive patients (**Figure 2**, list in **Table S1**). Several osteogenic biomarkers, including SOST, DKK1, PHOSPHO1 and SERPINH1, were upregulated in chemoresistant patients. The upregulated DEGs were also enriched in terms including ossification, collagen fibril organization, and the VEGF signaling pathway. The downregulated DEGs were enriched in several terms related to the immune response. Collectively, based on bulk RNA-seq data, chemoresistant osteosarcoma was characterized as expressing osteogenic-related gene sets (24), with relatively weaker expression of immune response-related gene sets.

**Table 2:** Patient clinical manifestations.

	Total (n=43)	Chemosensitive patients*^†^* (n=18)	Chemoresistant patients*^‡^* (n=25)	p value
Age (years)				0.54
Median(P_5_-P_95_)	14.81 (9.72-28.90)	15.65 (9.69-29.46)	14.37 (9.62-26.53)	
Sex				0.77
Female	18 (41.9%)	8 (44.4%)	10 (40.0%)	
Male	25 (58.1%)	10 (55.6%)	15 (60.0%)	
Metastasis at diagnosis				0.50
Metastasis	12 (27.9%)	6 (33.3%)	6 (24.0%)	
Localized	21 (72.1%)	12 (66.7%)	19 (76.0%)	
Status				
PFS^§^	23 (53.5%)	14 (77.8%)	9 (36.0%)	0.007
OS^¶^	29 (67.4%)	15 (83.3%)	14 (56.0%)	0.059

^†^necrotic rate ≥90% or Huvos stage III & IV indicated chemosensitive osteosarcoma.

^‡^necrotic rate <90% or Huvos stage I & II indicated chemoresistant osteosarcoma.

^§^PFS is “Progression-free survival”.

^¶^OS is “Overall survival”.

### scRNA-Seq Data Quality Control and Annotation

**Figure 3:**
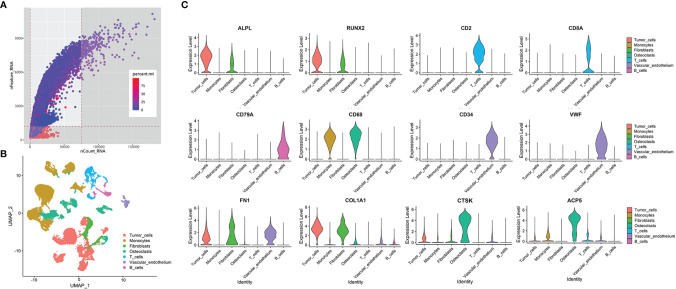
Overview of data processing and cell annotation in scRNA data. **(A)** Dot plot of data quality control in scRNA data. The threshold was set as nFeature_RNA > 1200, 500<nCount_RNA<75000. Area that was not covered by gray is the corresponding value range. The color of the dot represents the percentage of mitochondrial gene expression, whose cutoff value was less than 25%. **(B)** UMAP plot showing clusters of 7 main cell types from 6 osteosarcoma scRNA-seq datasets. **(C)** Violin plots exhibiting the expression of representative markers across diverse cell types. ALPL and RUNX2 are osteoblastic tumor markers. CD2 and CD8A are T-cell markers. CD79A is a B-cell marker. CD68 is a monocyte marker. CD34 and VWF are vascular markers. FN1 and COL1A1 are fibroblast markers. CTSK and ACP5 are osteoclast markers.

The 10x Genomics scRNA-seq data of 6 prechemotherapy osteosarcoma samples in GSE162454 were used in our study (9). For scRNA data, the sequencing depth is shown in **Figure 3A**. We used the following data: (1) features > 1200; (2) total RNA counts between 500 to 75000; and (3) percent of mitochondrial gene expression <25%, and 37321 eligible cells were standardized and normalized. First, the expression profile was normalized using the *LogNormalize* method, and 5000 hypervariable gene features were identified using the *variance-stabilizing transformation (VST)* method. Then, single-cell data were scaled and regressed against patients and percent mitochondrial gene expression. Next, the scRNA profile was subjected to *principal component analysis (PCA)* and *linear dimensionality reduction* with 50 presumptive principal components (PCs). Finally, we set 20 dimensions of reduction, which could exhibit 61.4% cumulative percent of variation and 0.01% change of variation between neighboring PCs, to identify clusters of cells by *shared nearest neighbor (SNN)* modularity optimization. The cells were classified into clusters based on the *UMAP algorithm* (**Figure 3B**, cluster markers in **Table S2**). Based on marker genes (**Figure 3C**, listed in **Table 3**), the clusters were annotated into osteoblastic tumor clusters, immune cell clusters (including monocyte clusters, T-cell clusters and B-cell clusters) and stromal cell clusters (including fibroblast clusters, osteoclast clusters, and vascular endothelial cell clusters).

**Table 3:** The canonical markers for the cell types in osteosarcoma tissues.

Cell cluster	Marker genes
Osteoblastic tumor cells	RUNX2, ALPL, IBSP
Monocytes	CD14, CD68, CD74
T cells	CD2, CD3, CD4, CD8A, IL7R
B cells	CD19, CD79A
Osteoclasts	ACP5, CTSK, MMP9
Fibroblasts	DCN, FN1, COL1A1
Vascular endothelium	CD34, VWF

### Tumor Cell Annotation Related to Drug Resistance

**Figure 4:**
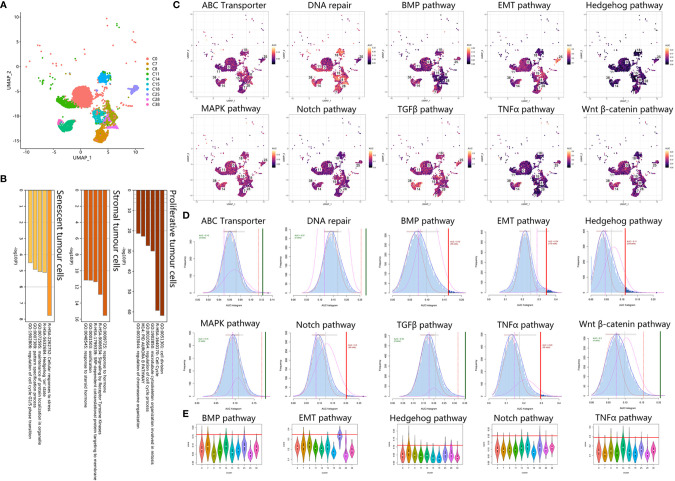
Tumor cells exhibited chemoresistant expression. **(A)** UMAP plot showing tumor clusters. **(B)** Bar plot for the top-ranked gene set enrichment in markers in proliferative, stromal and senescent tumor cells. **(C)** Scatter plot showing the distribution of AUCell scores in chemoresistant-related gene sets. **(D)** AUCell score distribution curves of chemoresistant related gene sets. The vertical line represents the cutoff of the AUCell score. The green vertical line indicates that the score was relatively homogeneous, and the red line indicates that the score could be divided into 2 groups, with high expression above and low expression below. As a result, BMP, EMT, Hedgehog, Notch, and TNFα are relatively heterogeneous in osteosarcoma. **(E)** Violin plots exhibiting the AUCell score of chemoresistant expression across tumor cell types. The red horizontal line is the cutoff value.

To identify tumor heterogeneity in osteosarcoma, we further investigated tumor cell clusters. Ten tumor clusters with 11777 cells were annotated in the previous step (**Figure 4A**). First, based on bulk RNA results, we focused on osteogenic-related expression among tumor cells (**Figure 4B**). Osteosarcomagenesis has been reported to be closely associated with the osteoblastic lineage and shows osteogenic differentiation-related activity in proliferation, ECM secretion and ossification induction (25, 26). We noticed that C11, C18 and C38 strongly upregulated proliferative expression, including DNA replication, chromosome organization and miotic expression. C0, C8, C14, C15 and C25 are stromal tumor cells that were characterized for their osteogenic function through the expression of ECM secretion and ossification induction. These stromal tumor cells also showed stimulation with several growth factors, such as PDGF, VEGF, EGF, and IGF, and activated downstream signals. C7 and C28 were identified as senescent tumor cells. They were characterized as weakly osteogenic activity but enriched in expression related to cellular stress and senescence.

Second, we analyzed chemoresistance-related expression in tumor cells. Using *AUCell* scoring, we noticed that the chemoresistance-related gene sets were all expressed in tumor clusters (**Figures 4C–E**). Among tumor clusters, the difference in chemoresistance-related expression was major in stemness-related expression, including EMT, Notch, TNFα, Hedgehog, and BMP gene sets, when the ABC transporter, DNA repair, TGFβ, Wnt β-catenin and MAPK gene set scores were relatively uniform. Based on the AUCell score threshold, the high EMT, TNFα, and Hedgehog cells were mostly concentrated in C7, C14, and C25, but high Notch and BMP cells were discretely distributed among tumor clusters. Together, these results suggested that C14 and C25 met the above two chemoresistant points and were characterized as osteogenic cancer stem cell (CSC)-like tumor cells.

### Differentiation Trajectory of Chemoresistant Tumor Cells

**Figure 5:**
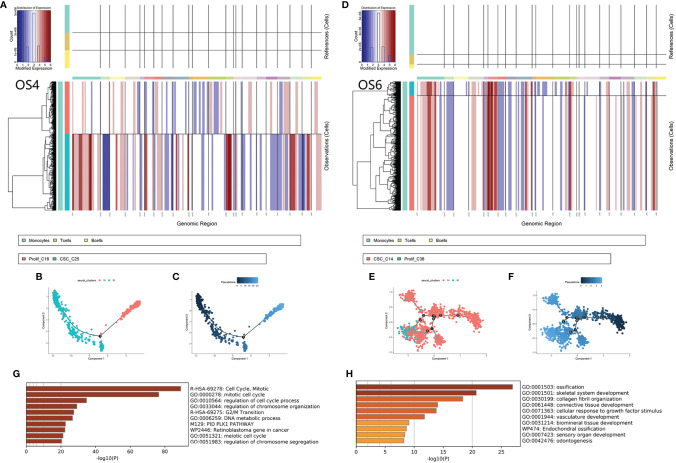
Differentiated trajectory of chemoresistant tumor cells. **(A, B)** Chromosomal CNV plots of tumor cells in OS4 (C18, C25) and OS6 (C14, C38). The above box was the control group. The lower box shows the tumor groups. The horizontal axis was segmentate as a chromosome. Red indicates amplification, and blue indicates censoring. As a result, C14 and C25 showed lower CNVs than C38 and C18. **(B–F)** Scatter plot showing the pseudotime trajectory from C25 to C18 **(B, C)** and from C14 to C38 **(E, F)**. **(G)** Bar plot for the top-ranked gene set enrichment in upregulated genes along the differentiated lineage. **(H)**. Bar plot for the top-ranked gene set enrichment in common marker genes in chemoresistant tumor cells.

C14 originated from OS4 patients. C25 originated from OS6. First, we calculated the CNV score of each cell to identify the change in chromosomes and inferred the differentiated trajectory among OS4 and OS6 tumor cells (**Figures 5A, D**). Based on *inferCNV*, we found that C14 and C25 exhibited lower CNV levels than C38 and C18 in OS4 and OS6, respectively (C14 vs. C38: 0.23 vs. 0.48; C25 vs. C18: 0.36 vs. 0.40). The results indicated that C14 and C25 had a lower degree of differentiation than C38 and C18.

Then, we applied *pseudotime analysis* among tumor clusters in OS4 and OS6 (**Figures 5B, C, E, F**). The *pseudotime* result also supported that tumor cells from C14 and C25 could differentiate to C38 and C18. A total of 217 genes were consistently involved in the differentiation from C14 and C25 to C38 and C18 and were strongly associated with mitosis and DNA replication. Based on the TRRUST database, the expression of these genes was closely regulated by transcription factors, including E2F1 and TP53. A total of 330 genes were common markers of C14 and C25 and were associated with the regulation of EMT and osteoblast differentiation. As a result, differentiated trajectory analysis supported the role of C14 and C25 in CSCs in osteosarcoma. The common markers between C14 and C25 suggested that tumor cells could be transmitted from CSCs into proliferative stages through active osteoblastic differentiation-like expression (**Figures 5G, H**).

### Nontumor Cellular Components Surrounding Chemoresistant Tumor Cells

**Figure 6:**
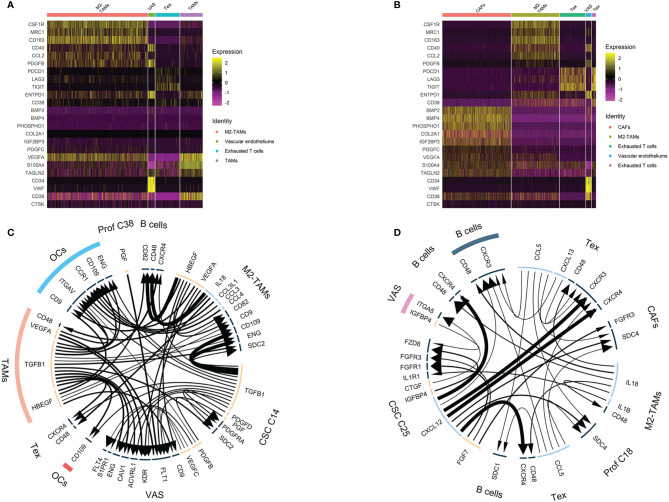
Nontumor cell and ligand–receptor interactions in chemoresistant osteosarcoma. **(A, B)** Heatmap showing TAM, exhausted T cell, CAF and tumor vascular cell marker expression. Colors from yellow to purple indicate the relative expression levels from high to low. **(C, D)** Ligand–receptor interaction plot in OS4 **(C)** and OS6 **(D)**. The arrow direction indicates the direction of the ligand–receptor interaction, in which the arrow is the receptor and the nock is the ligand. The thickness of the line indicates the relative expression levels from high to low. As a result, C14 and C25 showed a hub role between the tumor and osteosarcoma environment.

As we were aware, the nontumor cellular component accounted for more than half in the osteosarcoma environment (63.72% in OS4, 80.65% in OS6). This encouraged us to investigate diverse nontumor cellular components surrounding chemoresistant tumor cells, including immune cells and stromal cells (**Figures 6A, B**).

For immune clusters, we identified monocytes, T cells and B cells in the previous step. We noticed that monocyte clusters actively expressed markers of M2-TAM polarization, such as CCL2, CD40, CSF1R, CD163, IL10 and TGFβ1 (27). It has been reported that M2-TAMs can suppress the local immune response and maintain stemness in osteosarcoma (27). In OS6, T cells showed exhausted CD8 T-cell characteristics that upregulated inhibitory receptors, including PDCD1, LAG3 and TIGHT (28). The exhausted CD8 T cells and M2-TAMs together displayed multifaceted immunosuppressive signals within the osteosarcoma microenvironment.

For stromal clusters, we identified fibroblasts, vascular endothelium and osteoclasts in the previous step. We found fibroblasts expressing the CAF markers PDPN, S100A4 and TAGLN2 (29). The CAF cluster markers indicated that they had 3 functions in the osteosarcoma environment. First, it helped suppress local immunity through PDPN and S100A4 maintenance of M2 macrophage infiltration (30, 31). Second, it also played roles in the development of the osteogenic matrix by secreting collagen fibrils and ossification inducers, such as PHOSPHO1, BMP2, and BMP4. Third, it secreted rich growth factors, such as PDGFC, VEFGA, and IGF2BP, and stimulated cell growth. Vascular endothelial markers were strongly associated with blood vessel development and endothelial proliferation, which indicated active tumor angiogenesis in osteosarcoma.

Collectively, both the nontumor immune clusters and stromal clusters participated in the malignant progression of osteosarcoma by suppressing local immunity and promoting angiogenesis, osteogenic formation and CSC stemness. This result further supported that OS4 and OS6 were consistent with chemoresistant osteosarcoma in bulk RNA analysis.

### Ligand–Receptor Interactions in the Osteosarcoma Environment

Using *iTALK* analysis, the LR interactions among tumor clusters, immune clusters and stromal clusters in OS4 and OS6 were identified (**Figures 6C, D**, listed in **Table S3**). First, tumor clusters received immune and stromal cluster stimulation. C14 and C25 were stimulated with growth factor interactions such as PDGFD : PDGFRA, IGFBP4:FZD8, and TGFB1:SDC2. This also supported previous results that C14 and C25 activated growth factor signals. Additionally, it showed an IL1B:IL1R interaction with TAMs and was related to increased chemoresistance in osteosarcoma (32).

Second, tumor clusters affected both immune and stromal cells. C14 and C25 play main roles in secreting tumor-derived cytokines and growth factors. In OS6, C25 actively secreted FGF7 and CTGF, which promoted CAF growth and tumor angiogenesis, and CXCL12, which interacted with CXCR4 to block and deplete the T-cell response (33). In OS4, C14 secretes TGFB1, which is known for its important role in bone remodeling and regulation of the local immune response. It interacts with macrophages to promote M2-TAM polarization (34). It regulates the T and B immune response through the TGFB1: CXCR4 interaction (35). It mediates osteoclast activation in bone remodeling and tumor angiogenesis (36, 37). Together, we noticed that C14 and C25 acted as a bridge between tumor components and nontumor components. On the one hand, it receives messages from the environment that promote tumor growth and transform into the proliferative stage. On the other hand, it delivers diverse cell factors to immune and stromal cells, promoting tumor angiogenesis and bone remodeling and suppressing local immunity.

### Establishment and Validation Of the Chemoresistance Risk Model

**Figure 7:**
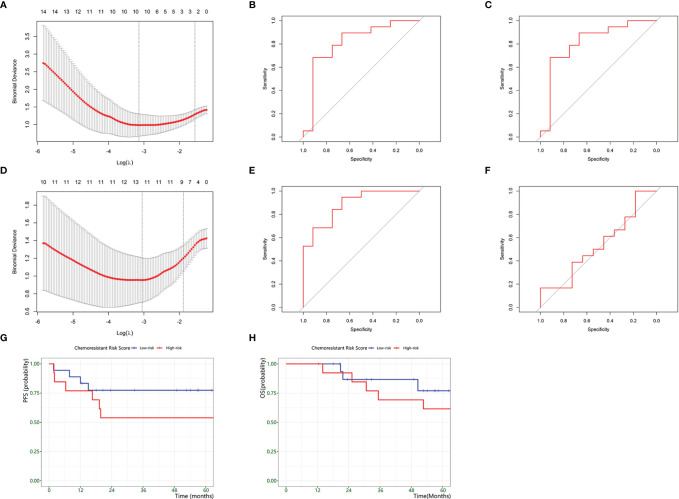
Development of a Chemoresistant Risk Scoring Model. **(A–C)** establishment of scRNA-based Chemoresistant Risk Scoring Model. **(A)** Chemoresistant expression feature selection in the LASSO model. **(B)** ROC curves to assess the accuracy of the scRNA-based chemoresistant risk scoring model to predict chemoresistance in the training groups. **(C)** ROC curves to assess the accuracy of the scRNA-based chemoresistant risk scoring model to predict chemoresistance in the training groups. **(D–F)** establishment of a bulk DEG-based chemoresistance risk scoring model. **(D)** Chemoresistant expression feature selection in the LASSO model. **(E)** ROC curves to assess the accuracy of the bulk DEG-based chemoresistant risk scoring model to predict chemoresistance in the validation groups. **(F)** ROC curves to assess the accuracy of the bulk DEG-based chemoresistant risk scoring model to predict chemoresistance in the validation groups. **(G)** Kaplan–Meier plot estimate of progression-free survival of patients by chemoresistant risk scores. **(H)** Kaplan–Meier plot estimate of progression-free survival of patients by chemoresistant risk scores.

Based on the above results, we identified chemoresistant C14 and C25 as being closely consistent with chemoresistant osteosarcoma characteristics in bulk RNA analysis. Thus, we explored the clinical application of their expression patterns in evaluating chemoresistance risk in osteosarcoma. The TARGET-OS series was used as the training set, and GSE33382 was used as the validation set (**Table 4**). First, we used univariate analysis to narrow down 1729 markers to 33 upregulated genes in chemoresistant osteosarcoma. Then, we selected 10 chemoresistant risk-related genes based on *LASSO algorithms* (**Figures 7A**, **Table 5**). Ultimately, the formula of the *chemoresistant risk score* was *Y*=−36.36+0.110∗*ADAMTS*2+0.042∗*SPAG*16+0.124∗*CGREF*1+0.328∗*JTB*+0.083∗*ENPP*2+0.528∗*ACP*1+1.485∗*NPM*1+0.759∗*CTSF*+0.045∗*MPP*6+1.109∗*PARD*6*G The chemoresistant Risk Score* was reliable and robust for predicting the chemoresistant risk of osteosarcoma based on *ROC curves* (*AUC_train_* = 0.82; *AUC_valid_* = 0.84 (**Figures 7B, C**).

**Table 4:** Patient clinical manifestations in the establishment and validation of the chemoresistant risk scoring model.

	Training cohort	Validating cohort
Dataset	TARGET-OS (n = 31)	GSE33382 (n = 29)
Age (years)		
Median (P_5_-P_95_)	15.10 (9.72-30.44)	16.00 (4.92-23.67)
Sex		
Female	12 (38.7%)	9 (31.0%)
Male	19 (61.3%)	20 (69.0%)
Chemotherapy respond^†^		
Sensitive	12 (38.7%)	11 (37.9%)
Resistant	19 (61.3%)	18 (62.1%)
Status		
Progression free survival	19 (61.7%)	Metastasis in 5yrs: 19 (65.5%)
Overall survival	23 (74.2%)	–

^†^necrotic rate ≥90% or Huvos stage III & IV indicated chemosensitive osteosarcoma; necrotic rate <90% or Huvos stage I & II indicated chemoresistant osteosarcoma.

**Table 5:** Chemoresistant risk-related genes based on the LASSO algorithm.

Gene name	Protein name
ADAMTS2	A disintegrin and metalloproteinase with thrombospondin motifs 2
SPAG16	Sperm-associated antigen 16 protein
CGREF1	Cell growth regulator with EF hand domain protein 1
JTB	Jumping translocation breakpoint protein
ENPP2	Ectonucleotide pyrophosphatase/phosphodiesterase family member 2
ACP1	Adipocyte acid phosphatase
NPM1	Nucleophosmin
CTSF	Cathepsin F
MPP6	M-phase phosphoprotein 6
PARD6G	Partitioning defective 6 homolog gamma

Meanwhile, we compared the power between the above scRNA-based risk model and the classic DEG-based risk model (**Figure 7D**). The DEG model was constructed by repeating the above steps, and the formula was *Y*=−5.712+0.920∗*PARD*6*G*+0.486∗*NPM*1+0.852∗*ATIC*+0.434∗*PDCD*4−1.560∗*EPS*8−0.335∗*PHF*19−0.304∗*ITGAL*−0.339∗*FOXM*1+0.029∗*EFHD*1−0.019∗*TPP*2−0.195∗*PDCD*1+0.290∗*ACP*1 . *AUC_train_* was 0.89, and *AUC_valid_* was 0.54 (**Figures 7E, F**). The results supported that the scRNA-based risk model was more robust and avoided overfitting than the classic DEG-based risk model.

Furthermore, we investigated the prognostic prediction of the *chemoresistant risk scoring model*. Using *ROC curves*, we found that -11.48 was the optimal cutoff value for *the chemoresistant risk score*, where the *AUC* value reached a maximum of 0.80. Therefore, we could divide the patients into a high chemoresistant risk *Score* ≥ -11.48 and a low chemoresistant risk (*Score* < -11.48). We explored the prognostic capability of *the chemoresistant risk score* of osteosarcomas. The results showed that high-risk patients tended to suffer from earlier progression and a lower 5-year PFS rate, but the difference was not statistically significant (*p* = 0.36) (**Figures 7G, H**).

## Discussion

In osteosarcoma, the combination of chemotherapy and surgery is the cornerstone of treatment, and a good response to chemotherapy could further improve patient survival (1). However, predicting chemotherapy response and enacting timely adjustment before neoadjuvant chemotherapy remain challenges. Transcriptome sequencing is a feasible way to detect chemoresistant related cells in osteosarcoma biopsy samples and to predict chemotherapy response (6). As technology advances, scRNA-seq has enabled us to obtain higher-resolution data from osteosarcoma tumor cells and explore the heterogeneous chemoresistant tumor environment. In our study, we aimed to reveal chemoresistance-related expression profiles and establish a chemoresistance risk score model for osteosarcoma patients based on a combination of scRNA-seq and bulk RNA-seq data.

Using bulk RNA-seq, we revealed the general glance in chemoresistant osteosarcoma: active osteogenic expression and suppressive immune expression. Our chemoresistance landscape is consistent with previous reports suggesting that the immunosuppressive and rich ossific ECM microenvironment is a barrier that severely limits osteosarcoma patient survival (38, 39). However, it is known that the existing chemoresistant tumor cells play a core role in the construction of a chemoresistant environment when bulk RNA results provide only a view of average expression. The expression characteristics of chemoresistant tumor cells remain unclear.

scRNA sequencing enabled us to resolve gene expression at the individual cell level and has been widely investigated in solid tumors, including osteosarcoma (9, 10). This provided us with the chance to capture chemoresistant tumor cells according to Liu’s study (9). In this study, we obtained a comprehensive single-cell expression atlas of prechemotherapy osteosarcoma and annotated tumor cells, T cells, B cells, monocytes, fibroblasts, osteoclasts and vascular endothelium. The cell types were similar to those in Liu’s report, demonstrating the reliability of our data processing.

To recognize chemoresistant tumor cells, we reviewed previous literature and summarized key chemoresistant expression patterns, including ABC transporters, DNA repair, stemness expression (EMT, Wnt/β-catenin pathway, TGFβ pathway, TNFα pathway, MAPK pathway, Notch pathway, Hedgehog pathway and BMP pathway) (6). Combining this result with the active osteogenic function from bulk RNA results, we identified C14 and C25 as most fitting to the chemoresistant expression patterns among tumor cells. They showed both osteogenic functions, such as ossific ECM secretion, and were stimulated with growth factors and high stemness expression, such as activating EMT and the TNFα pathway.

In our study, we undertook a more thorough analysis of C14, C25 and their surrounding osteosarcoma environment. CNV estimation and pseudotime analysis illustrated a differentiated trajectory from C14 and C25 to proliferative C38 and C18. In addition, we noticed that the transmission came through upregulating osteoblastic differentiation-like expression. Studies have revealed that osteoblastic differentiation is closely associated with osteosarcoma tumorigenesis (25, 26). This result supported the osteogenic CSC roles of C14 and C25.

CSCs make up the dominant roles in osteosarcoma chemoresistance, and the tumor environment acts as fertile soil for CSCs (40, 41). We found that C14 and C25 played a bridge role between tumor cells and the cellular environment. On one side of the bridge, C14 and C25 receive growth signals from the environment. The results showed that the growth factor LR interacts with PDGFB : PDGFRA, IGFBP4:FZD8, and TGFB1:SDC2 with TAMs and tumor vascular cells. C14 and C25 possessed FGF7, IGFBP4, and PDGFD paracrine functions, further promoting tumor growth and differentiation to the proliferative stage. On the other side of the bridge, C14 and C25 transmit messages to nontumor cells to shape an angiogenic, immunosuppressive and ossific environment. C25 actively secretes FGF7 and CTGF, which promote CAF growth and tumor angiogenesis, and CXCL12, which interacts with CXCR4 to block and deplete the T-cell response (33). C14 secretes TGFB1, which participates in bone remodeling and the regulation of the local immune response (42). As a response, we noticed that exhausted CD8 T cells and M2-TAMs together displayed multifaceted immunosuppressive signals within the osteosarcoma environment, which could suppress the local immune response and maintain stemness in osteosarcoma (27, 28). For stromal clusters, CAFs help to maintain M2 macrophage infiltration and play roles in developing osteogenic matrix when the tumor vasculature shows actively proliferative and angiogenic function. Collectively, the results supported OS4 and OS6 fitting with characteristics of chemoresistant osteosarcoma in bulk RNA analysis.

The detection of existing chemoresistant tumor cells is a feasible method for chemoresistant risk assessment (7). In the era of precision medicine, the RNA sequencing-based risk model is one of the most widely applied risk assessment tools to evaluate the chemoresistant risk for a particular patient and to plan individualized treatment. During the past years, several bulk RNA-seq-based signatures have been identified for chemoresistance prediction in osteosarcoma and other solid tumors (43, 44). However, these models sometimes failed to show synchronously consistent predictive performance in both the training and validation groups. This could be explained as bulk RNA-based tumor markers sometimes being drowned by environmental noise, since bulk RNA results reflect average expression and ignore the heterogeneity of tumor components in the solid tumor environment. Our results also showed a similar phenomenon in which the traditional DEG-based model had unsatisfactory performance in validating the environment (*AUC_train-bulk DEGs_* =0.89; *AUC_valid-bulk DEGs_* = 0.54). As technology advances, single-cell sequencing can be used to identify the expression patterns in individual cell clusters. A recent study attempted to apply scRNA-seq-based signatures in prognostic prediction in ovarian cancer (45). The scRNA-seq-based signatures revealed the roles of macrophages in ovarian cancer progression and showed robust performance in predicting patient prognosis. In this way, we drew our inspiration and presented a single-cell sequencing-assisted strategy for establishing an osteosarcoma chemoresistant risk prediction model. As stated above, the chemoresistant tumor cells C14 and C25 play central roles in the chemoresistant osteosarcoma environment, and we assessed chemoresistance by detecting their existence. Therefore, using the scRNA-based chemoresistant markers in C14 and C25, we constructed a chemoresistant risk score model in prechemotherapeutic osteosarcoma, which presented satisfying and stable performance in inferring the likelihood of chemoresistance in osteosarcoma patients (*AUC_train_* = 0.82; *AUC_valid_* =0.84).The scRNA-based model showed comparable performance with the classic bulk DEG-based model in the training environment (*AUC_train-scRNA_* = 0.84; *AUC_valid-bulk DEGs_* =0.54).

In our study, some limitations must be recognized. First, due to the absence of a histologic response in the scRNA-seq dataset, we identified chemoresistant tumor cells based on findings from bulk RNA-seq and lacked follow-up of the scRNA-seq patients. Experimental verification of drug resistance in chemoresistant tumor cells and chemoresistant marker expression in osteosarcoma tumor tissue were necessary to clarify the association between histologic response and the chemoresistance risk score. Second, our model lacks consideration of the adjustment strategy in the high chemoresistant risk group. Further improvement of the model could focus on experimental and clinical exploration for the latent adjustment strategy of chemotherapy in high chemoresistant risk patients. Third, the small sample size weakened the validation of the effectiveness of the model. The model performance and prognostic prediction capability required a larger sample size.

## Conclusion

In this study, we provided a perspective for understanding chemoresistant osteosarcoma tumor cells based on combining scRNA and RNA data. Using bulk RNA data, we found that osteogenic expression was upregulated in chemoresistant osteosarcoma compared to that of chemosensitive osteosarcoma. Then, we transferred the bulk RNA findings to single cell sequencing data and identified osteosarcoma tumor clusters C14 and C25 that show osteogenic cancer stem cell expression patterns, which fit chemoresistant characteristics. In addition, it possessed bridge roles in interactions with other clusters. On the one hand, they received various growth factor stimulators and could potentially transform into a proliferative state. On the other hand, they promote local tumor angiogenesis, bone remodeling and immunosuppression. Furthermore, we established a chemoresistant risk score model to evaluate the chemoresistant risk for prechemotherapeutic patients with osteosarcoma. The model had a satisfying discriminatory ability and was more robust in predicting chemoresistant risk in both the training and validation groups than classic bulk RNA-based models. This approach could potentially assist with prechemotherapeutic assessment and personalized adjustment strategies in osteosarcoma.

## Data Availability Statement

Publicly available datasets were analyzed in this study. The datasets for this study can be found in the TARGET-OS and GEO databases. The original contributions presented in the study are included in the article/**Supplementary Material**. Further inquiries can be directed to the corresponding authors.

## Author Contributions

GL, LB and HX conceived the studies. ZZe, LW, ZD, ZC designed the research process and were major contributors in writing the manuscript. JX collected and assembled the data in the TARGET-OS and GEO databases. GR, WZ, YC, YH, ZZh, WQ, HR, ZQ participated in software support and data analysis. All authors read and approved the final manuscript.

## Funding

The study was supported by the Science and Technology Program of Guangzhou, China (201707010089), Medical Science and Technology Research Foundation of Guangdong Province, Guangzhou, China (A2021371), Funding of Basics and Application Basics of Guangzhou (202102020096), and Funding of Regenerative Medicine and Health Laboratory of Guangdong (1102101201).

## Conflict of Interest

The authors declare that the research was conducted in the absence of any commercial or financial relationships that could be construed as a potential conflict of interest.

## Publisher’s Note

All claims expressed in this article are solely those of the authors and do not necessarily represent those of their affiliated organizations, or those of the publisher, the editors and the reviewers. Any product that may be evaluated in this article, or claim that may be made by its manufacturer, is not guaranteed or endorsed by the publisher.
